# Announcement: 3rd European Conference on Bio-Inorganic Chemistry

**DOI:** 10.1155/MBD.1996.55

**Published:** 1996


					UROBIC 3
August 4-10, 1996, Noordwijkerhout, The Netherlands
3rd EUROPEAN CONFERENCE ON
BIO-INORGANIC CHEMISTRY
INTERNATIONAL ADVISORY BOARD
L. Banci, Florence, Italy
I. Bertini, Florence. Italy
F. Gonzalez-Vilches, Seville, Spain
M. Ao de la Rosa, Seville, Spain
J. Reedijk, Leiden, The Netherlands
R. Thorneley, Brighton, UK
C.Veeger, Wageningen. The Netherlands
NATIONAL ORGANIZING COMMITTEE
Chairman C. Veeger
Telephone +31 317 482868
Telefax +31 317 484801
Secretary: M.C. Feiters
Treasurer: W. R. Hagen
Members: S. P. J. Albracht, G. W. Canters, B. Hessen, J. Reedijk, I. M. C. M. Rietjens, H. Schoemaker,
C. Veeger, R. Wever
The organizing committee cordially invites you to participate in EUROBIC 3, the 3rd European Conference on
Bio-inorganic Chemistry. The Conference will be held at the Leeuwenhorst Congress Centre, Noordwijkerhout
(near Leiden, about 20 km from Amsterdam), The Netherlands, in August 4-10,1996. The Conference is or-
ganized under the auspices of the Royal Netherlands Chemical Society (KNCV).
SCOPE OFTHE CONFERENCE
The Conference will focus on all actual themes from current Bio-inorganic Chemistry, such as:
Structure-function relationships in Metalloenzymes
Coordination compounds in Medicine
Models for Metalloprotein Active Sites
Spectroscopy of bio-inorganic systems and models
and others
LANGUAGE
English is the official language of the Conference.
SCIENTIFIC PROGRAMME
The programme will consist of invited plenary lectures, invited lectures in 2 parallel mini-symposia, and con-
tributed poster presentations.
YOUNG SCIENTISTS
The organization stimulates the participation of young scientists, it is intended that they will be partially
refunded.
PLENARY LECTURES
The following speakers have accepted the invitation to give a plenary lecture:
L. Que, Minneapolis, USA
L. Dutton, Philadelphia, USA
M Vol'pin, Moskow, Russia
C. D. Garner, Manchester, UK
B. Meunier, Toulouse, France
I. Bertini, Flor.ence, Italy
P. Kroneck, Konstanz, Germany
G. Wachtrshauser, Munich, Germany
PROCEEDINGS
Proceedings, including abstracts of plenary lectures lectures from mini-symposia and poster presentations,
will be available at the conference. Contributors will be asked to submit their abstracts in camera-ready
format a few months before the conference.
REGISTRATION
The final procedure for registration will be given in the second circular which will be distributed in January
1996. The costs of full board and registration will be approximately Hf. 1400,-.
PRELIMINARY REGISTRATION
Receipt of the 2nd circular can be ensured by writing to
Prof. C. Veeger,
Department of Biochemistry,
Wageningen Agricultural University,
Dreyenlaan 3,
NL-6703 HA Wageningen,
The Netherlands
Conference E-mail Address: EUROBIC@SECR.BC.WAU.NL

				

